# Assessing Mineral Content and Heavy Metal Exposure in Abruzzo Honey and Bee Pollen from Different Anthropic Areas

**DOI:** 10.3390/foods13121930

**Published:** 2024-06-19

**Authors:** Federica Flamminii, Ada Consalvo, Angelo Cichelli, Alessandro Chiaudani

**Affiliations:** 1 Department of Innovative Technologies in Medicine and Dentistry, University “G. d’Annunzio” of Chieti-Pescara, Via dei Vestini, 66100 Chieti, Italy; angelo.cichelli@unich.it (A.C.); achiaudani@unich.it (A.C.); 2 Center for Advanced Studies and Technology (CAST), “G. d’Annunzio” University of Chieti-Pescara, Via Luigi Polacchi, 11, 66100 Chieti, Italy; ada.consalvo@libero.it

**Keywords:** honey, bee pollen, ICP-MS, heavy metal, risk assessment, environmental impact

## Abstract

Honey and bee pollen offer potential health benefits due to their nutrient and bioactive molecules, but they may also harbor contaminants such as heavy metals. This study aimed to assess the content of different metals, including Mg, Al, K, Ca, V, Cr, Mn, Fe, Co, Ni, Zn, Cu, As, Rb, Sr, Cd, Cs, Tl, Pb and U, in honey and bee pollen collected from different Abruzzo region (Italy) areas (A1, A2, A3, A4), characterized by different anthropic influences described by Corine Land Cover maps. Differences were observed in the mineral and heavy metal content associated with the influence of biotic and abiotic factors. Honeys were found to be safe in regard to non-carcinogenic risk in all the consumer categories (THQ_m_ < 1). A particular carcinogenic risk concern was identified for toddlers associated with Cr (LCTR > 1 × 10^−4^) in A1, A2 and A3 apiaries. Pb and Ni represent potential non-carcinogenic and carcinogenic health risks in children and adults due to bee pollen consumption, showing high values of THQ_m_ and LCTR. The results suggest the advantages of utilizing bee products to screen mineral and heavy metal content, providing valuable insights into environmental quality and potential health risks.

## 1. Introduction

Since ancient times, throughout history, honey has accompanied human beings, serving as a vital food source and a key element in religious, mystical, and medicinal practices [1].

Honey, produced by *Apis mellifera* bees, comes from the nectar of flowers, plant secretions, and aphids’ honeydew. Bees collect, transform, dehydrate, store, and mature it in the honeycomb [2]. It is rich in nutrients, predominantly carbohydrates, which make up about 75% of its composition, with glucose and fructose being the main sugars (85–95%). Additionally, honey contains traces of organic acids, enzymes, amino acids, and pigments. The water content ranges from 10 to 25%, while minerals, varying by the honey’s origin, constitute 0.04%–0.2%. Key minerals include potassium (K) (up to 70%), calcium (Ca) and sodium (Na), magnesium (Mg), iron (Fe), manganese (Mn), and zinc (Zn), with the average contents exceeding 1 mg kg^−1^ [3,4,5,6].

Along with its nutritional properties, honey brings several functional and health benefits (antimicrobial, antioxidant, anti-inflammatory, antidiabetic, wound healing, anticancer, anti-proliferative, immunomodulatory effects, gastrointestinal tract diseases, cardiovascular effects, ophthalmology) well documented and described by Aga and coauthors [7].

The worldwide production of honey in 2021 reached 1772 M ton^−1^, and about 22% was produced in Europe. In Italy, 23.000 t of honey were produced in 2022, and about 3% of this amount (690 t) was produced in the Abruzzo region [8].

Bee pollen is harvested by honeybees from plant flowers and enriched with salivary enzymes and nectar to obtain small granular-looking grains (bee pollen) that are transported into the apiary [9]. It contains carbohydrates (13–55%), proteins (10–40%), lipids (1–13%) and fibers (0.3–20%), with a moisture content varying from 4 to 8%. Additionally, bee pollen has a high mineral content (2.5–6.5 g/100 g), predominantly potassium (K) (about 60% of total mineral content), along with magnesium (Mg), sodium (Na) and calcium (Ca), ranging from 10% to 20% [10,11,12,13,14,15]. Bee pollen is also rich in secondary metabolites, including biotin, folic acid, carotenoid pigments, niacin, phytosterols, polyphenols, thiamine, tocopherol, flavonoids, sterols, terpenes, vitamins, enzymes, and coenzymes. Consuming daily doses of 20–40 g of bee pollen can provide recommended daily intakes (RDIs) for various elements at notably high levels.

Bee pollen is a natural strength supplement to the body’s immune and physiological systems, making it attractive for use in the diets of children and adults suffering from certain avitaminoses and loss of appetite. It improves blood supply to the nerve tissue, powers mental performance, and reduces the state of fatigue while having a positive effect on the liver, heart, prostate, and allergy diseases. The primary consumers of bee pollen include advocates of health-conscious and environmentally friendly lifestyles, as well as the elderly, due to its antioxidant and other therapeutic effects [10,11,12,13].

Mineral elements contained in honey and bee pollen could be both essential and non-essential to human biological functions. An inadequate dietary deficiency of the essential mineral elements results in a variety of diseases or syndromes; conversely, these mineral elements can become harmful in excessive amounts [14,15]. Non-essential minerals can exert toxicity, even at low concentrations, and can affect the level of the essential elements in the body [16,17]. The essential elements include macrominerals (sodium, magnesium, phosphorous, sulfur, chlorine, potassium, and calcium) and trace elements (silicon, vanadium, chromium, manganese, iron, cobalt, nickel, copper, zinc, selenium, molybdenum and iodine).

The macrominerals, with an average content exceeding 1 mg kg^−1^ [18], are responsible for the maintenance of the ionic balance of structural skeletal compounds, amino acids, and nucleic acids. Trace elements have several physiological and biochemical functions for the correct cellular metabolism, influencing the circulatory system and reproduction and composing structural proteins, hormones, and key enzymes, e.g., zincin, iron in hemoglobin, and selenium in glutathione peroxidase enzyme [19,20,21,22].

Certain non-essential elements, such as aluminum, vanadium, arsenic, rubidium, strontium, cadmium, cesium, thallium, lead, and uranium, may contaminate honey and pollen, and among the most potentially toxic are heavy metals characterized by a high atomic weight (over 63.5 and with a specific gravity higher than 5.0). The major elements included in this class are as follows: Pb, Cd, Co, Cr, Cu, Fe, As, Ni, Zn, and Hg [23]. In general, they are found naturally on the Earth’s crust, but their growing utilization results in an increase in metallic substances in both the terrestrial and aquatic environments [24]. The primary pollution sources are the metal-based industries, leaching of metals from landfills, waste dumps, excretion, livestock and chicken manure, runoffs, automobiles, and roadworks. The use of pesticides, insecticides, and fertilizers in agriculture are the secondary source of heavy metal pollution [25]. Also, natural causes, such as volcanic activity, metal corrosion, metal evaporation from soil and water and sediment re-suspension, soil erosion, and geological weathering can increase heavy metal pollution.

Heavy metals interact with nuclear proteins together with DNA, causing site-specific direct and indirect damages. In the first case, conformational changes occur to the biomolecules, while the second is a result of the production of reactive oxygen and nitrogen species such as hydroxyl and superoxide radicals, hydrogen peroxide, nitric oxide, and other endogenous oxidants. These toxic elements can lead to acute damage to many vital organs, like the kidneys, liver, brain, etc. In addition, prolonged exposure can trigger blood pressure alteration, anxiety, and passivity disorders. According to the US Environmental Protection Agency (USEPA) and the International Agency for Research on Cancer (IARC), arsenic, cadmium, chromium, lead, and mercury are the most dangerous metals and are also classified as either “known” or “probable” human carcinogens [21]. Chromium and nickel can exert toxicity, affecting the respiratory system and inducing carcinogenesis, allergies, infection diseases, and intestinal microbes [26,27]. The consumption of arsenic element in food products leads to skin lesions and cardiovascular disturbances, while cadmium is also responsible for placental damage, prostate cancer, and renal lesions; moreover, ingestion of mercury can cause cerebral palsy and mental retardation. Children are vulnerable to lead, with it particularly affecting their brain and peripheral nervous system development, while hypertension and kidney damage were observed in adults upon its prolonged consumption [22,28,29]. The latter is the only one to date that has a maximum limit in honey set by law, set at 0.10 mg kg^−1^ wet weight, as established by the Commission Regulation [30].

In general, the content of individual elements can vary considerably among the different honey and pollen taxa. This variation depends on the plant species that bees forage as well as on the landscape and the different morphological characteristics that influence the chemical composition of the ground that surrounds the beehives, which, in turn, is influenced by the levels of environmental pollution [31,32,33]. The need to monitor and protect the environment more carefully and detect the sources of pollution have become highly topical issues. In recent years, environmental monitoring with bees has assumed greater importance due to its characteristics of management simplicity and cost-effectiveness. Due to their extensive flight range of approximately 1.5 to over 3 km from their hive, covering an estimated area of about 7–28 km^2^ (700–2800 ha) [13,34], bees serve as dynamic environmental sensors, unlike many other largely immobile bioindicators [35,36,37]. The mortality rates of these bioindicators correlate with the levels of environmental pollution, making them valuable for detecting traces of harmful pollutants such as agricultural pesticides, antibiotics from human and livestock sources, heavy metals, radionuclides, pathogenic microorganisms, and other contaminants [38]. Moreover, bees function as “biofilters”, mitigating contamination levels in nectar during honey production, even in environments with high pollution levels [39,40,41,42].

As a result, honey and pollen, being susceptible to environmental influences, can be significant sources of chemical contaminant exposure, posing potential public health concerns. However, because bee pollen undergoes less of a transformation by bees, it tends to provide a more accurate reflection of environmental contamination [43]. In this regard, some authors have suggested that pollen may serve as a superior bioindicator of environmental pollution compared to honey, which is most effective as a bioindicator in cases of significant contamination [44].

The aim of the present study was to examine the mineral composition of polyfloral honey collected from various areas within the Abruzzo region. Additionally, the investigation was extended to bee pollen obtained from the same hives, aiming to evaluate the impact of the “natural” filtration process carried out by the bees. The selected sampling areas were categorized based on their urban, agricultural, and natural environments, representing a territory model. Furthermore, based on the elemental composition data, the study conducted a risk assessment to evaluate both the carcinogenic and non-carcinogenic risks associated with the ingestion of honey for toddlers, children, adolescents, and adults as well as the ingestion of pollen for children and adults, highlighting the diverse implications associated with their consumption.

## 2. Materials and Methods

### 2.1. Sample Collection

**Figure 1:**
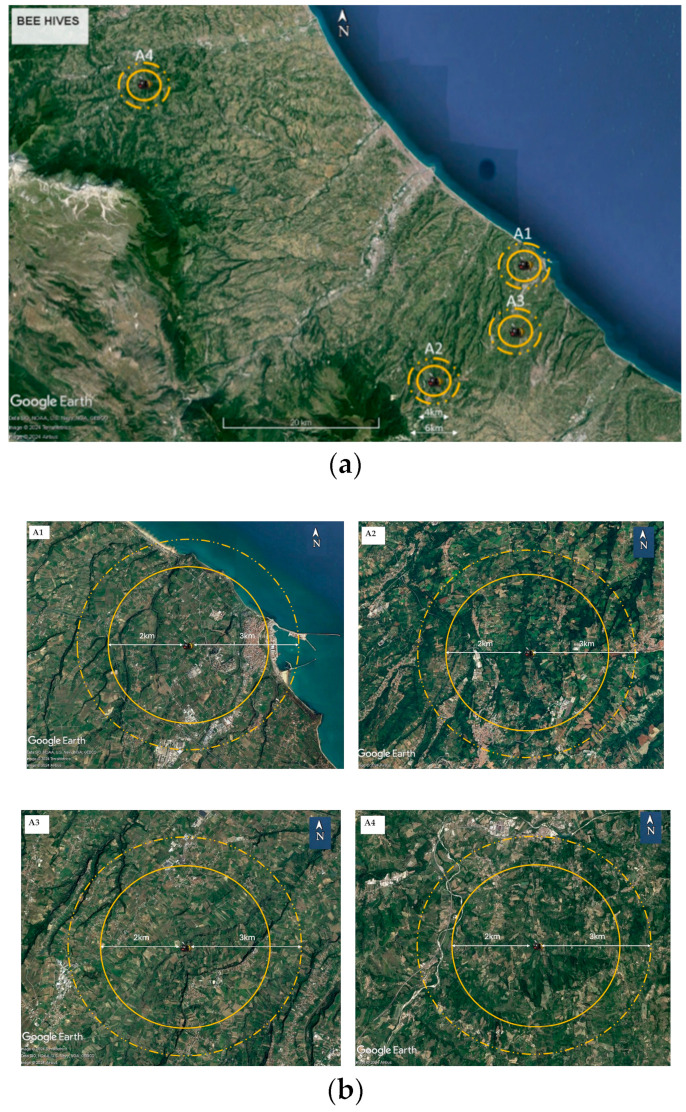
(**a**) Geographical location of the beehives in Abruzzo region and (**b**) the flight area of each beehive (**A1**–**A4**). The images are adapted from those obtained from Google Earth Image © Airbus 2024 Image © TerraMetrics 2024.

Eight honey samples and nine bee pollen samples were collected in the following four suburban areas of the Abruzzo region: the Chieti province (Ortona, Filetto and Frisa municipalities, Italy) and the Teramo province (the Capsano district in the Penna S. Andrea municipality, Italy). Apiaries were coded as follows: A1: Ortona, A2: Filetto, A3, Frisa, A4, S. Andrea. Figure 1 shows the geographical locations of the sampling sites (Figure 1a) and the flight area of the beehives (Figure 1b).

Honey samples were gathered during the periods of May–June 2019 and June–July 2020, whereas bee pollen samples were collected in July 2020 and September 2020. Fresh polyfloral honey and bee pollen were directly procured from beekeepers and subsequently stored in laboratory-grade bottles at a temperature of −20 °C until analysis.

### 2.2. Corine Land Cover Use of Soil

To deepen the spatial pressure of land use on forage areas, Google Earth and Corine Land Cover 2018 (CLC) project map information tools were used. The Corine Vector soil data viewer codes appropriately different land-use types, with a 25 ha/100 m minimum mapping unit (Copernicus Land Monitoring Service [45]. The description of the codes is reported in the Supplementary Materials (Table S1). The soil use maps were produced around the beehives for a flight area of 12.5 km^2^ (2 km from their hive).

### 2.3. Sample Preparation

Honey and pollen samples were digested using a previously described procedure [46,47]. Acidic digestion of 0.1 g of pollen samples was performed in sterile polystyrene tubes (15 mL, BD FalconTM, BD Biosciences, Franklin Lakes, NJ, USA) by adding 0.750 mL 69% (*v*/*v*) HNO_3_, heating at 60 °C O/N, and subsequently by adding 0.500 mL of 30% (*v*/*v*) H_2_O_2_ with final heating at 60 °C for 8 h. Acidic digestion of 1 g of honey samples was performed by adding of 1 g of 18.2 MΩ cm^−1^ deionized water and 2 mL of 69% (*v*/*v*) HNO_3_ heating at 60 °C for 8 h. The digested pollen and honey samples were diluted to a final volume of 12 mL and 8 mL, respectively, with 18.2 MΩ cm^−1^ deionized water, and they were analyzed by Inductively Coupled Plasma Mass Spectrometry (ICP-MS). The external standard method was applied for quantification, and we later verified the accuracy of the method with fortification experiments and the calculation of recovery values. An internal standard correction was performed by online addition of an internal standard solution of Rh and Y (50 μg L^−1^) in a T-piece. Duplicate analysis was performed for each sample. The HNO_3_ concentration of external standard solutions was accurately matched to the final concentration of HNO_3_ in the samples (i.e., 3.8%).

### 2.4. ICP-MS Analysis of Elements

ICP-MS analyses were performed by using a 7500A ICP quadrupolar mass spectrometer (Agilent Technologies, Palo Alto, CA, USA) fitted with an ASX-510 autosampler (CETAC, Omaha, NE, USA) and a peristaltic pump. A Babington nebulizer with a Scott spray chamber (Agilent Technologies) was used for sample introduction. Detailed operating conditions and instrumental parameters are given in Table S2. The optimization of ICP-MS was carried out to obtain maximum signal intensities for 7Li, 89Y, 140Ce and 205Tl using a tuning solution while keeping the formation of oxides 140CeO^+^/140Ce^+^ and doubly charged species Ce^2+^/Ce^+^ ratios below 1% and 2%, respectively. Pollen and honey samples were analyzed for Mg, Al, K, Ca, V, Cr, Mn, Fe, Co, Ni, Zn, Cu, As, Rb, Sr, Cd, Cs, Tl, Pb and U. The external standard method was applied for quantification, after which we verified the accuracy of the method with fortification experiments and calculation of recovery values. An internal standard correction was performed by the online addition of an internal standard solution of Rh and Y (50 μg L^−1^) in a T-piece. Duplicate analysis was performed for each sample. The HNO_3_ concentration of external standard solutions was accurately matched to the final concentration of HNO_3_ in the samples (i.e., 3.8%). Data analysis was performed using ChemStation software (version G1834B) (Agilent Technologies).

### 2.5. Risk Assessment

The non-carcinogenic and carcinogenic health risks through the consumption of honey and bee pollen were assessed according to the estimated daily intake (*EDI*), target hazard quotient (*THQ*), hazard index (*HI*) and lifetime cancer risk (*LTCR*) [48,49,50].

#### 2.5.1. Non-Carcinogenic Risk

The *THQ* is the probable non-carcinogenic risk for orally ingested elements; it is defined as the ratio of the daily oral intake to the oral reference dose with the following equation, as suggested by the United States Environmental Protection Agency (US EPA):

$$THQ=\frac{EDI}{RfDm}$$

The estimate daily intake (*EDI*) value was calculated according to the formula suggested by USEPA and other authors [51,52,53].

EDI=(C×IR×EF×TE)/(BW×AT)

where *C* is the concentration of each potentially toxic element (PTE) detected in the samples (mg/kg), *IR* is the intake rate of honey and bee pollen (kg/day), *EF* is the exposure frequency to the contaminant (350 day/year), *TE* is the total exposure, and *AT* is the average lifetime time for non-carcinogenic risk (*TE* × 365 day/year). The dates related to *BW*, *AT*, *TE* and *IR* that are related to different groups and used for the assessment of *EDI* are reported in Table 1.

RfD_m_ is the oral reference dose (mg/kg_bw_/day) (Table 2). Given the challenges in setting a reliable threshold for lead (Pb) according to the USEPA, this study relied on the RfD_Pb_ (reference dose for lead) proposed by previous research as a suitable alternative [60,61,62,63].

A *THQ_m_* (dimensionless) >1 entails a high non-carcinogenic risk, as the adverse health effect is considerable, while, if *THQ_m_* is <1, it is generally presumed to be safe for the risk of non-carcinogenic effects.

The cumulative risk arising from the dietary exposure to all elements in the same foodstuff, in our case honey or bee pollen, was assessed through the Hazard Index (*HI*). representing the cumulative sum of *THQ_m_* values for each element and calculated as follows:

$$HI=\sum_{m}THQm$$

A *HI* > 1 entails a high potential health impact implication, at the opposite a *HI* < 1 indicates that there is no apparent health impact due to the metals considered. A serious chronic health impact has been suggested for *HI* > 10 [2].

#### 2.5.2. Carcinogenic Risk

The *LCTR* is the carcinogenic effect related to the ingestion of food contaminated by Ni, Cr, Pb, As, and Cd [51].

LTCR=EDI×CSF

*CSF* represents the cancer slope factor (mg/kg_bw_/day)^−1^ that estimates the probability of developing cancer due to the ingestion of Ni, Cr, Pb, As, and Cd. The CSF_Cd_ proposed (Table 1) was previously used by other authors [62,64,66].

The US EPA considers an *LTCR* (dimensionless) >1 × 10^−4^ as an unacceptable risk in regard to developing cancer over a human lifetime. *LTCR* values between 1 × 10^−6^ and 1 × 10^−4^ are considered to be an acceptable range for carcinogenic risk. The Canadian Safe Environments Directorate (2010) proposes the value of 1 × 10^−5^ as the maximum safety threshold for the risk of developing cancer [71].

The cumulative cancer risk is the risk estimation due to exposure to multiple carcinogenic elements and is calculated as:

$$LTCRtot=\sum_{k=1}^{n}LTCRk$$

where *LTCRk* is the life time cancer risk for the cancer element *k*.

### 2.6. Statistical Analysis

Data were expressed as mean ± standard deviation. One way ANOVA and a Kruskal−Wallis test were used to investigate significant differences among samples where the a confidence level was held at 95%. Principal component analysis (PCA) and hierarchical cluster analysis (HCA) were performed with honey and bee pollen datasets. Data analysis was performed using XLSTAT software (version 2023.3.1) (Addinsoft SARL, New York, NY, USA) and ClustVis, a web tool freely available at http://biit.cs.ut.ee/clustvis/ (accessed on 1 February 2024) [72].

## 3. Results

### 3.1. Use of Soil and Characterization of the Flight Areas (CLC)

**Figure 2:**
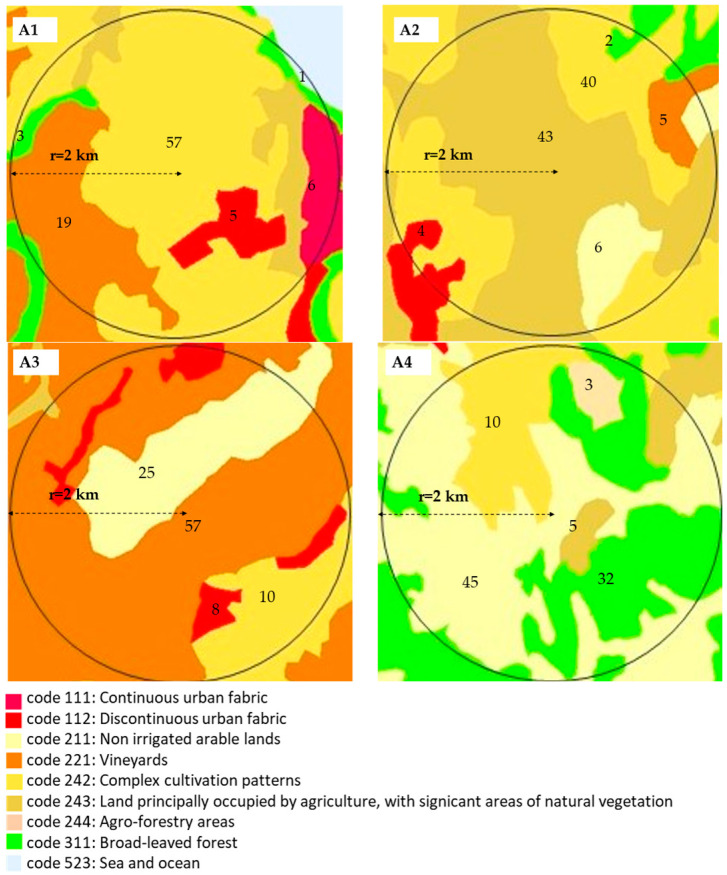
Corine Land Cover use in the beehive areas (r = 2 km) of A1: Ortona (CH), A2: Filetto (CH), A3: Frisa (CH), A4: S. Andrea (TE). Numbers in figure represent the percentage of use by the different types of area, as coded.

Bees, flying in their extensive foraging areas, come into contact with air, water, and soil, potentially picking up contaminants like PTEs and transferring to their hives and hive products. Therefore, investigating soil usage is crucial. The study examined four specific areas (Figure 2) based on information provided from the CLC project map, modified by ArcGis 10.6 software (Redlands, CA, USA), and significant differences among the specific uses of soil were highlighted. The Apiary 1 (A1) flight area was the one mainly characterized by the presence of a continuous (code 111) and discontinuous (code 112) urban fabric at around 11%, followed by complex cultivation and vineyards at around 76%. The Apiary 2 (A2) flight area was represented mainly by the presence of agriculture, with significant areas of natural vegetation (code 243) and complex cultivation patterns (code 242) at around 83% and a small portion of discontinuous urban fabric and vineyards. The Apiary 3 (A3) flight area was covered mainly by vineyards (≈57%), a portion of non-irrigated arable land (≈25%), generally under a crop rotation system, and a smaller area of discontinuous urban fabric and complex cultivation patterns (≈18%). The Apiary 4 (A4) flight area was characterized by the significant presence of vegetation formation composed principally of trees (code 311) at around 32%, followed by non-irrigated arable land (≈45%), complex cultivation systems (≈10%), and agro-forestry and natural vegetation areas at around 8%. In brief, the A1 apiary was situated in the most heavily anthropic environment, whereas the A4 flight area was positioned within the Natural Regional Reserve of Castel Cerreto (Teramo, Abruzzo), representing the least anthropized environment. A2 and A3 exhibited intermediate levels of anthropization.

### 3.2. Mineral Contents of Honey and Bee Pollen Samples

The complex interplay of bees’ environment, vegetation, floral sources, climate, and geographical traits gives rise to unique varieties of honey. The resulting mineral profile serves as a crucial tool for evaluating its nutritional value, identifying its geographic origin, and detecting environmental contamination by heavy metals [6]. The concentrations of the twenty elements detected in multifloral honey samples obtained from the four different areas are reported in Table 3. Except for the A1 area, all the other samples were harvested both in 2019 and 2020.

Concerning the essential elements, the most abundant macrominerals were K, Ca, and Mg, with mean values of 590.6, 39.6 and 20.2 μg g^−1^, respectively. Despite the great variability, all the element results were in line with the content observed by different authors for honeys from the center and south of Italy, indicating K to be the most abundant mineral in honey, followed by Ca, Na, and Mg [6,18,73,74,75,76]. Conversely, iron content was not detected (<LOD) in honey samples.

Trace elements such as Mn, Co, Cu, Zn, Rb, Sr, Al, and Tl showed mean values of 0.244, 0.002, 0.249, 1.086, 0.407, 0.184, 1.11, and 0.0009 μg g^−1^, respectively. Among these, either Zn, in sample A4 (with a mean value of 2.13 μg g^−1^), and Al, in sites A2 and A3 (with means values of 2.31 and 1.49 μg g^−1^, respectively), showed the highest values.

With regard to heavy metals, the mean concentrations were of 0.004 μg g^−1^ (As), 0.0231 μg g^−1^ (Ni), 0.0317 μg g^−1^ (Pb) and 0.222 μg g^−1^ (Cr), while Cd was not detected (<LOD) in the samples. Interestingly, the levels of Pb and Cr were significantly higher (*p* < 0.05) in A1, A2, and A3 with respect to A4, denoting an anthropic pollution in the first three areas, as observed previously. Indeed, Cr has been reported to be very widespread in the environment, and, in absence of metallurgical and chemical manufacturing industries located near the hives, it could be transferred to different distances due to the wind action, meteorological factors, topography, and vegetation, which are strictly related to the long-transfer of the metal [35]. Pb is one of the most widespread environmental pollutants, and this is mainly attributed to internal-combustion engines [77]. Regarding the high affinity of Pb as an atmospheric particular matter, the presence of emission sources of particles like road asphalt and tires around the hive can cause honey to be contaminated with Pb. All the honey samples resulted within the legal limits for lead content (100 μg kg^−1^), considering that the highest level of Pb in honey samples was 53.5 μg kg^−1^, confirming the excellent quality of the analyzed honeys and the lower transfer capacity of the elements from the environment via bees to the final product. No significant differences were highlighted for nickel content (*p* < 0.05), while higher values (*p* > 0.05) were found for arsenic in the A2 and A3 areas, with mean values of 0.005 and 0.004 μg g^−1^ respectively.

The element contents found in this study aligned with the literature data previously obtained by other authors and reported in Table S3. However, it is possible to highlight the great variability in terms of qualitative and quantitative composition due to biotic and abiotic factors, such as the effect of anthropic pollution frequently reported by other authors, even within the same variety [78,79]. Furthermore, some research considered different or fewer elements than those observed in the present study. The values we found in this investigation were generally comparable to the values of honey originating from different Italian areas [40,80,81]. Furthermore, differences in analytical approach, including the methods of sample solubilization and determination techniques, may also affect the results [82].

The elemental composition of bee pollen is reported in Table 4. No significant differences (*p* < 0.05) were observed for Ca, V, Cr, Cu, As, Cd, Cs, Tl, and U among the four areas and between the two harvesting periods (July and October), with mean values of 1223, 0.055, 0.187, 12.95, 0.054, 0.040, 0.038, 0.032 and 0.031 μg g^−1^, respectively. Concerning the essential elements, despite significant differences among the samples (*p* < 0.05), the most abundant macrominerals were K, Ca, and Mg, with mean values of 5985, 1233, and 853 μg g^−1^, respectively. The same behavior was observed for the non-essential minerals, where the harvest area significantly influenced the elemental content of bee pollen (*p* < 0.05). This peculiar aspect highlights the difficult-to-compare literature data of bee pollen from both different Italian regions and foreign countries; therefore, for the sake of clarity, an exhaustive summary of bee pollen mineral composition was reported in Table S4. Overall, the results found in the present study are in accordance with the content reported by different authors [13,27,48,58,73,74,83].

Focusing on heavy metals, no significant differences (*p* < 0.05) were observed for Cr, As, and Cd, with mean values of 0.187, 0.054, and 0.040 μg g^−1^. Conversely, Ni and Pb highlighted significant differences (*p* < 0.05) among areas. Nickel had the lowest and the highest results, as seen in A2 (0.51 μg g^−1^) and A4 (1.56 μg g^−1^), respectively. Lead resulted significantly higher (*p* < 0.05) in the A1 area (7.1 μg g^−1^) than in A2, A3, and A4, where no differences were denoted (1.03, 1.19 and 0.06 μg g^−1^). Several studies support the toxic metal concentrations of bee pollen significantly depending on the degree of environmental pollution [11,32,84,85,86]. In the case of Ni, particularly high in A4, A1, and A3 (*p* > 0.05), it could be influenced by the natural geochemistry of soils [87,88], industrial processes, vehicle emissions, the combustion of fossil fuels, waste disposal, or the use of pesticides in agricultural practices [89].

**Figure 3:**
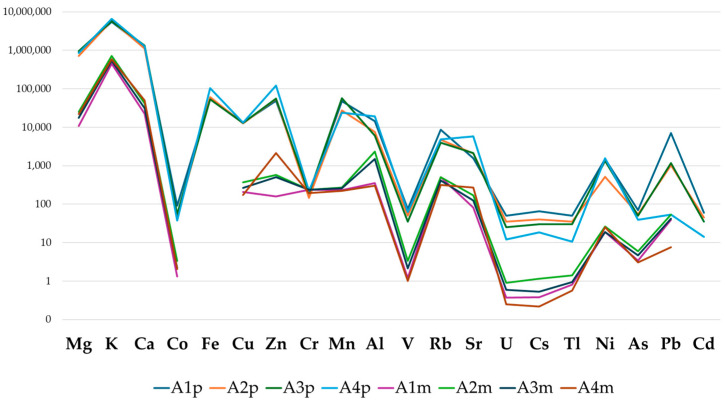
Elemental pattern of bee pollen (p) and honey (h) samples in the respective apiary (A1–A4).

Comparing the mean mineral content in bee pollen and honey (Figure 3), it was possible to highlight that the elemental profile exhibited similar geospatial trends or patterns associated with the same origin of the two beehive products. The detected honey concentration in the decreasing range (mean concentrations) was K > Ca > Mg > Zn > Fe > Mn > Cu > Al > Rb > Sr > Pb > Ni > Cr > Co > V>As > Cs > Tl > U, while in bee pollen it was K > Ca > Mg > Al > Zn > Rb > Cu > Mn > Cr > Sr > Pb > Ni > As > Co > V>Tl > Cs > U. Overall, the concentrations of bee pollens were 10–70 times higher compared to that in honey, similar to what has been observed by other authors, supporting the hypothesis of biological reduction in the levels of metals in the finished product. Indeed, this aspect is associated with the activity of bee enzymes during the honey elaboration process or with the presence of molecules such as gluconic and ascorbic acid, responsible for the chelation of elements and complex formations, leading to the absorption and accumulation of metals in specific body anatomic sections or excretion with feces rather than their accumulation in honey [90,91]. Conversely, differences in the pattern were denoted for Cr and Mn that showed a smaller (0.82) and a higher ratio (153), respectively.

As frequently mentioned, the mineral content of bee pollen and honey is strictly related to the vegetal species in terms of metabolism, physiology, and morphology, which influence the amount of elements in the different parts of the plant tissues, flowers included [89]. It was also observed that the capability of certain plants to concentrate pollutants can also affect their concentration in the honey sample. For example, honey obtained from the nectar of aromatic plants is characterized by a high concentration of heavy metals since they tend to concentrate pollutants more than herbaceous plants [75]. Furthermore, polluted bee pollen results in higher levels of metals than in honey, suggesting the potential use of such products as indicators of metal pollution in their areas of origin as well as of potential health risks [16].

### 3.3. Carcinogenic and Non-Carcinogenic Effects in Bee Pollen and Honey

The bioaccumulation of PTEs in a body fed by plants, feeds and animal-origin foods, as well as water, can contribute to a wide variety of adverse health effects, including organ damage, developmental alterations, and cancer [92]. Specific regulations regarding the presence of PTEs in honey and bee pollen are currently lacking. However, the Codex Alimentarius includes a stipulation that honey must be devoid of quantities of metals that could pose a hazard to human health.

In the present study, PTE accumulation rates and possible risk levels were estimated according to the daily honey and bee pollen consumption amount. For honey, more frequently consumed than bee pollen, the recommended daily dose is at around 10 g for toddlers, adolescents, and adults. In the case of bee pollen, the main consumers follow a health and environmentally conscious lifestyle, as well as the elderly, who use it due to its antioxidant and other therapeutic effects. Its recommended daily dose consumption was reported to range from 20 to 40 g for children and adults, respectively.

#### 3.3.1. Non-Carcinogenic Risk (*EDI*, *THQ_m_*, *HI*)

The *EDI* estimates the daily exposure level of the human population to toxic and potentially toxic elements through food consumption. The mean estimated daily intake (*EDI*) of the analyzed metals were assessed for toddlers, children, adolescents, and adults for honey, as well as for bee pollen in regard to children and adults, due to the poor information about bee pollen consumption in these categories. The total *EDI* rank of all metals for honey follows the decreasing order of toddlers (0.69 mg/day) > children (0.29 mg/day) > adolescents (0.15 mg/day) > adults (0.11 mg/day), while, for bee pollen, the trend is children (6.03 mg/day) > adult (4.52 mg/day).

The honey and bee pollen *EDI* ranks of individual metals for all groups follow the decreasing order of K > Ca > Mg > Al > Zn > Rb > Cu > Mn > Cr > Sr > Pb > Ni > As > Co > V>Tl > U, and K > Ca > Mg > Zn > Fe > Mn > Cu > Al > Rb > Sr > Pb > Ni > Cr > Co > V>As > Cd > Cs > Tl > U, respectively. Interestingly, the *EDI* related to the apiaries shows the rank order A2 > A4 > A3 > A1 for honey and A3 > A4 > A2 > A1 for pollen.

Overall, the *EDI* of each metal obtained is reported to be lower than the correspondent maximum tolerable daily intake for both honey and pollen.

**Figure 4:**
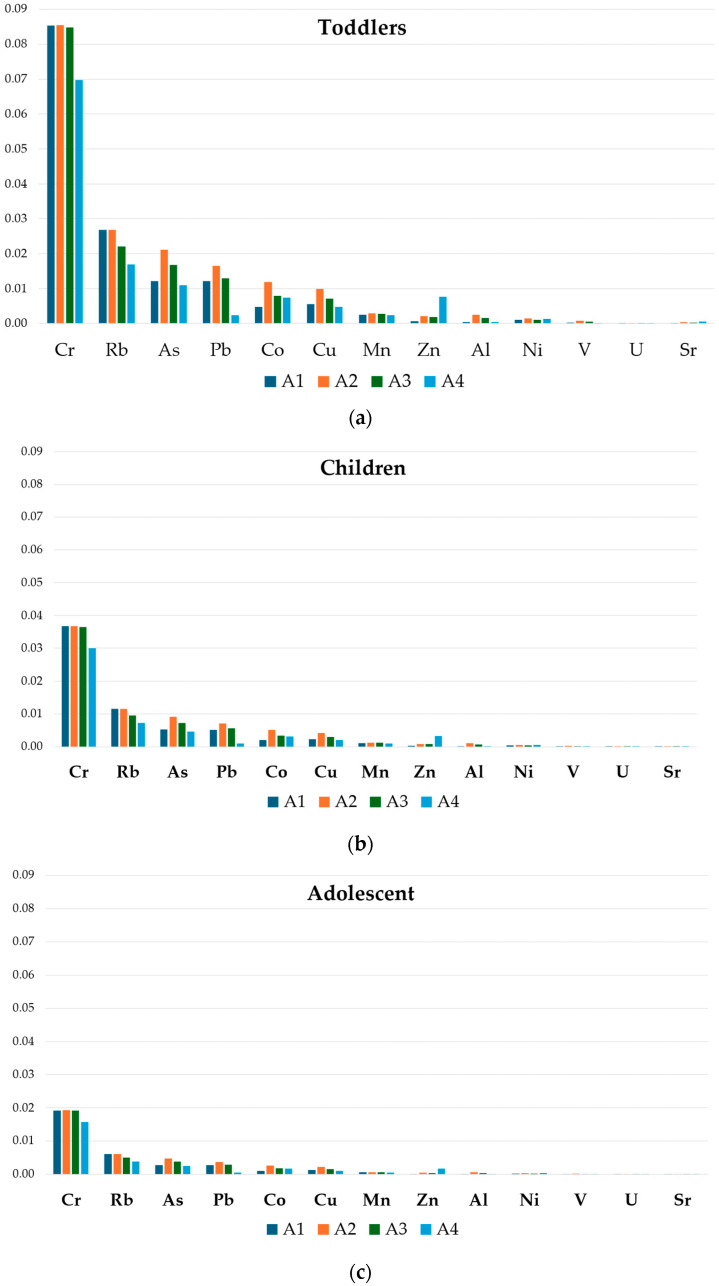

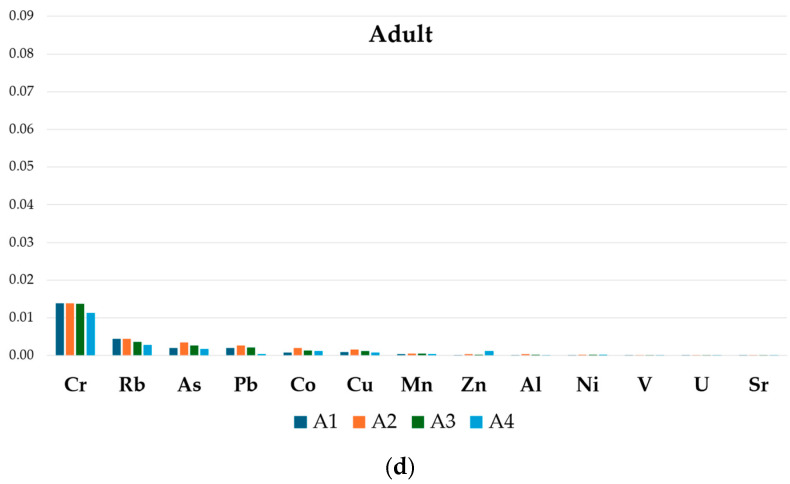
*THQ_m_* value for honey in toddlers (**a**), children (**b**), adolescent (**c**) and adult (**d**) in different apiaries.

**Figure 5:**
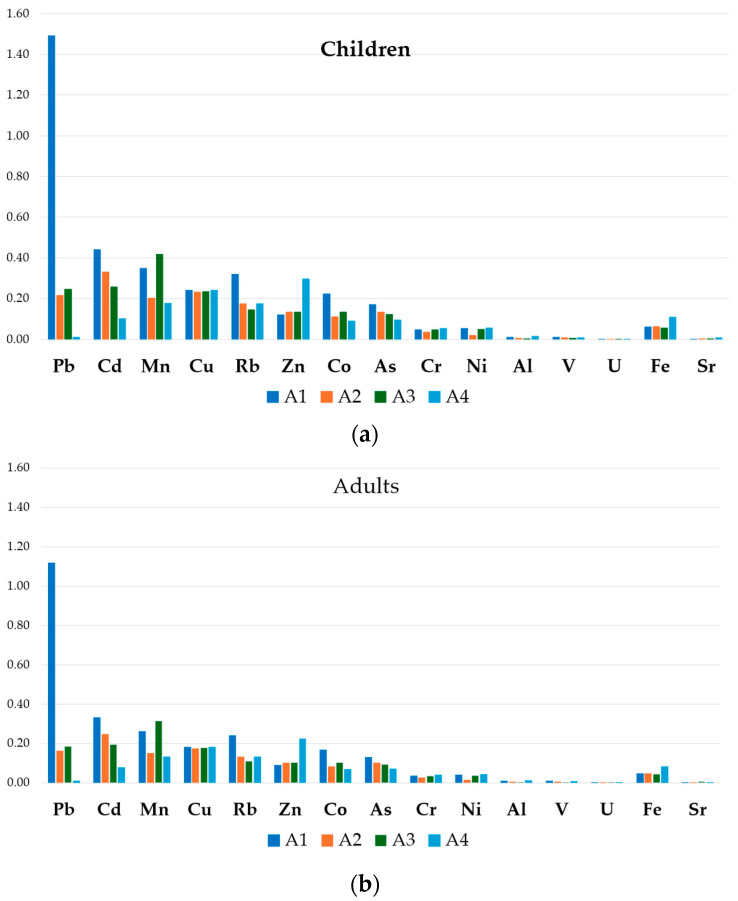
*THQ_m_* value for bee pollen in children (**a**) and adult (**b**).

The *THQ_m_* values for honey and bee pollen were reported in Figure 4 and Figure 5, respectively. For all the analyzed elements, the *THQ_m_* values in honey were below 1, suggesting that the exposed human population is supposed to be safe [48]. Conversely, in bee pollen samples, and mainly for those belonging to the A1 apiary, the *THQ_m_* value resulted above 1 for Pb for both the children and adult group, indicating a potential health risk associated with its consumption.

In the four apiaries, the honey average *THQ_m_* exposure values ranged from 8.5 × 10^−2^ (Cr) in toddlers (A1) to 1.4 × 10^−6^ (U) in adults (A4), while in bee pollen, the *THQ_m_* higher values ranged from 1.5 (Pb) in children (A1) to 2.2 × 10^−4^ (U) in adults (A4).

Considering all groups, the honey and bee pollen *THQ_m_* values of individual metals followed the decreasing order of Cr > Rb > As > Pb > Co > Cu > Zn > Mn > Ni > Al > V>U, and Pb > Cd > Cu > Mn > Rb > Zn > Co > As > Cr > Ni > Al > V>U, respectively.

**Figure 6:**
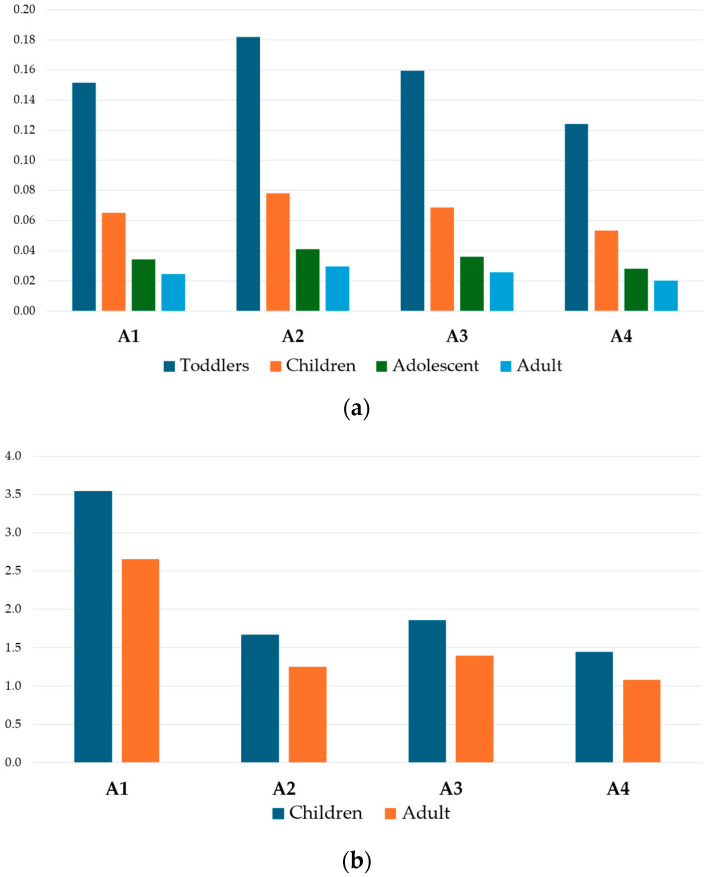
*HI* index for honey (**a**) and bee pollen (**b**) for different consumer categories and apiaries.

The sum of the *THQ_m_* values for each category, represented by the *HI* index, was reported in Figure 6. Honey samples (Figure 6a) showed values below the safety threshold (<1) for all consumer groups and apiaries, resulting in no health concerns. Contrarily, for bee pollen samples (Figure 6b), only the adult group in the A4 apiary presented a *HI* value below 1; therefore, the consumption of bee pollen belonging to the A1, A2, and A3 apiaries represented a health risk concern. For honey, the average *HI* risk rank, based on the consumer groups, was toddlers (0.154) > children (0.066) > adolescents (0.035) > adults (0.025), while for bee pollen it was children (2.1) > adults (1.5), higher than honey because of the greater metal concentrations.

The honey average *HI* rank order based on all apiaries corresponded to A2 > A3 > A1 > A4, while in bee pollen it was A1 > A3 > A2 > A4. For honey, the highest HI value occurred for toddlers in the A2 apiary (0.18), and it was the lowest for the adult group in the A4 apiary (0.020); for bee pollen, the highest *HI* value occurred for children in the A1 apiary (3.5) and the lowest occurred for the adult group in the A4 apiary (0.99). It was quite evident that the A4 apiary could be considered safer in terms of honey and bee pollen than the other sites.

**Figure 7:**
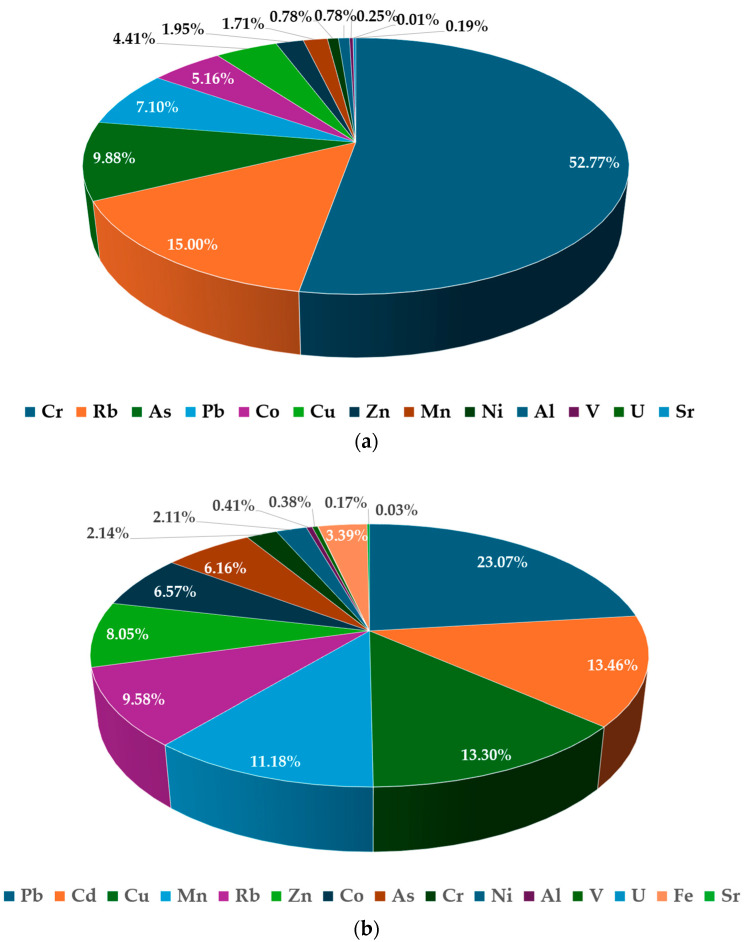
Contribution (%) of each metal to *HI* due to the consumption of honey (**a**) and bee pollen (**b**).

The average percentual contribution of PTEs, reported in Figure 7, highlighted that, in the case of honey consumption (Figure 7a), the 52.9% was accounted by Cr, followed by Rb (15%), As (9.9%), and Pb (7.1%), while the rest of the metals cumulatively accounted for only 15.1%. In the case of bee pollen consumption (Figure 7b), the main contribution was related to Pb (23.9%), followed by Cd (14%), Cr (13.8%), Mn (11.6%), Rb (9.9%), Zn (8.4%), Co (6.8%), As (6.4%), while the rest of the metals cumulatively accounted for 5.24%.

#### 3.3.2. Carcinogenic Risk (LCTR)

**Figure 8:**
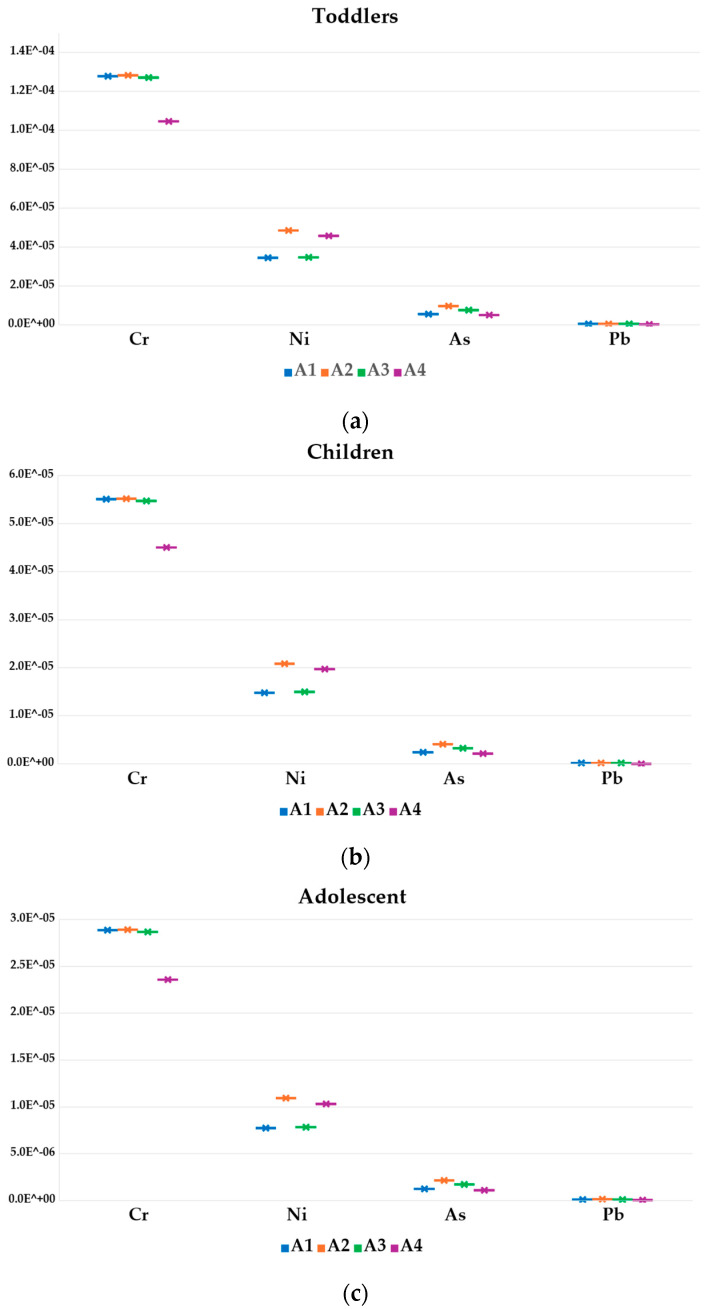

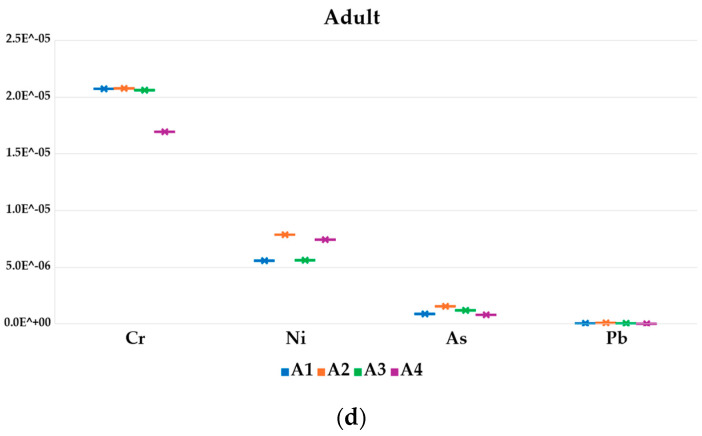
Lifetime cancer risk (*LCTR*) values based on carcinogenic elements exposure in toddlers (**a**), children (**b**), adolescent (**c**) and adults (**d**) in different apiaries.

The carcinogenic risk assessment (*LCTR*), calculated based on CSF values reported in Table 2, and particularly for Ni, Cr, Pb, As, and Cd, is shown in Figure 8. Concerning honey, *LTCR* value >1 × 10^−4^ was reported for Cr exposure in the toddlers category (Figure 8a) while *LTCR* value >1 × 10^−5^ was observed for children, adolescents, and adults (Figure 8b–d), following the apiary rank A2 > A1 > A3 > A4. Nickel *LTCR* values >1 × 10^−5^ were observed in toddlers and children, regardless the apiary, following the order A2 > A4 > A3 > A1, and for the adolescent category in apiaries A2 and A4.

**Figure 9:**
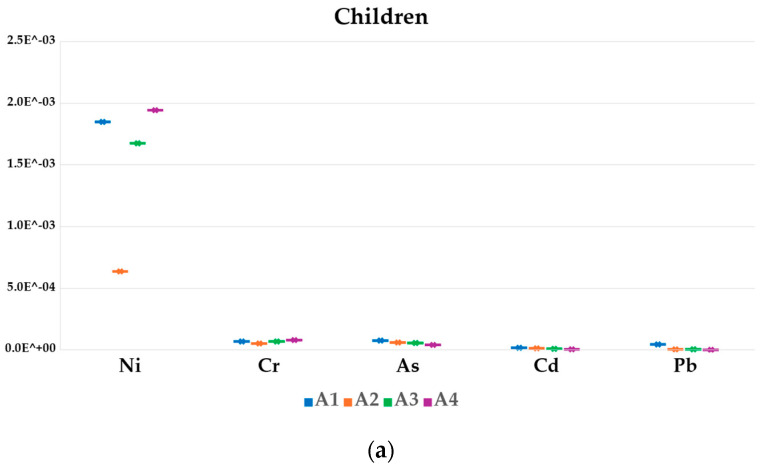

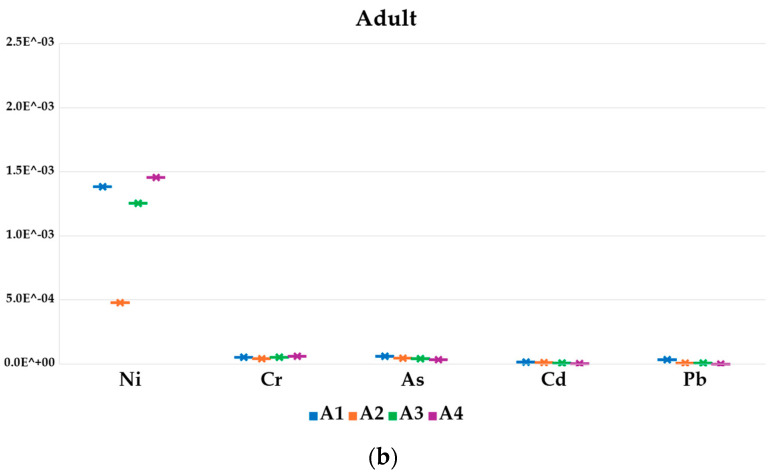
Lifetime cancer risk (LCTR) values based on carcinogenic element exposure in children (**a**) and adults (**b**) in different apiaries.

The *LTCR* related to bee pollen is reported in Figure 9. A Ni *LTCR* value >1 × 10^−4^ was observed for both children and adults, and for all the apiaries in the following order A4 > A1 > A3 > A2, *LTCR* >1 × 10^−5^ was observed for Cr, As, Cd, and Pb despite slightly different apiary ranks. In particular, Cr followed A4 > A1 = A3 > A2, while As followed an A1 > A2 > A3 > A4 order. The *LTCR* value for Cd was > 1 × 10^−5^, observed in children and adults with an apiary rank of A1 > A2, while *LTCR* >1 × 10^−5^ was recorded for Pb in adults, especially in the A2 apiary.

Several studies which characterized honey by values of *LCTR* included amounts of between 1 × 10^−5^ and 1 × 10^−4^, like those reported in this study [63,77,93,94,95,96].

In the case of bee pollen, *LCTRs* above 1 × 10^−5^ and 1 × 10^−4^ are described by [27,56].

The contribution of each element to *LCTRs* in honey and bee pollen is reported in Figure S1. Cr accounted for 71.8%, followed by Ni (24%), As (4%), and Pb (0.2%) in honey samples (Figure S1a). Concerning bee pollen (Figure S1b), Ni accounted for 90.9%, then Cr (4.1%), As (3.5%), Pb (4.1%), and Cd (9.6%).

**Figure 10:**
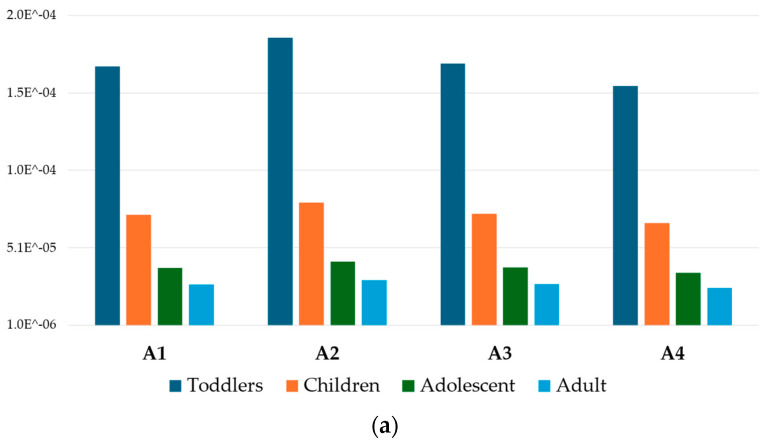

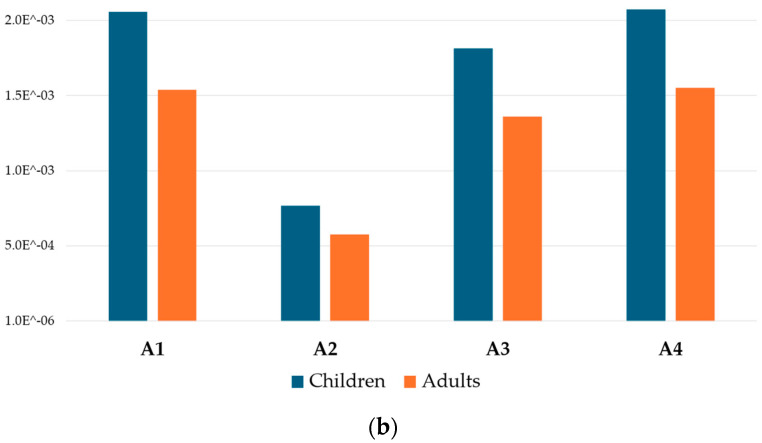
Cumulative lifetime cancer risk (LCTRtot) values based on carcinogenic element exposure in different consumer categories in honey (**a**) and bee pollen (**b**) in different apiaries.

The cumulative cancer risk (*LCTRtot*) is reported in Figure 10. With regard to honey, due to exposure to multiple carcinogenic elements, *LCTRtot* was >1 × 10^−4^ in the case of toddlers, while it was >1 × 10^−5^ for children, adolescents, and adults. Conversely, the *LTCRtot* value for bee pollen highlights values ranging from 1.3 × 10^−3^ to 7.7 × 10^−4^. The *LCTRtot* rank based on apiaries corresponds to A2 > A3 > A1 > A4 for honey, while for bee pollen it is A4 > A1 > A2 > A3.

### 3.4. PCA

**Figure 11:**
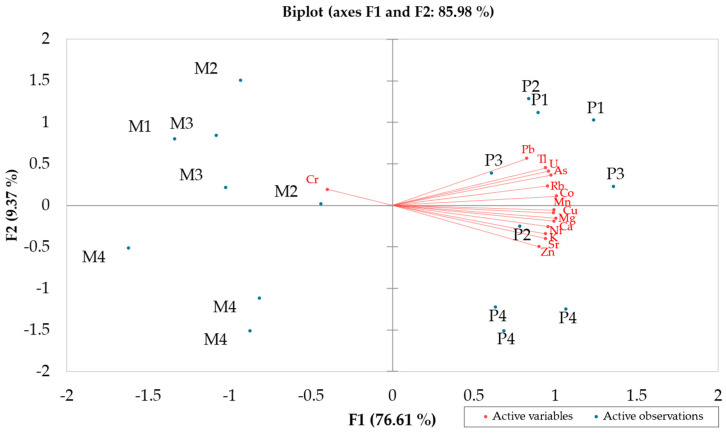
Principal component analysis (PCA) biplot showing the differentiation of the two bee product matrices by the first two principal axes.

The element content of honey and bee pollen samples were examined by PCA. Fe and Cd, non-detected in honey, were not considered. The biplot of loadings (variables) and score (observations), reported in Figure 11, highlighted a clear separation of the two macro samples, honey and bee pollen, along F1, which explains the 76.61% of the total variance (85.98%). Conversely, apiaries, regardless of the product, were well separated along the F2 component, since A1, A2 and A3 were located in the positive quadrants while A4 was in the opposite negative side. Further, bee pollens of A1, A2 and A3 were strongly correlated with most of the metal, except Cr, which was correlated with the honey belonging to the same apiaries. Observing the F2 component, apiary A4 was completely separated both for honey and bee pollen. In particular, bee pollen was found to be richer in Zn, opposite to A1, A2 and A3, which were richer in Pr, Tl, U, and As.

Results presented by PCA elaboration confirm those previously discussed; indeed, the products belonging to the apiaries A1, A2 and A3, unlike the A4 apiary, proved to be more contaminated with heavy metals such as Pb, Cr, As, and also Cd.

### 3.5. HCA

**Figure 12:**
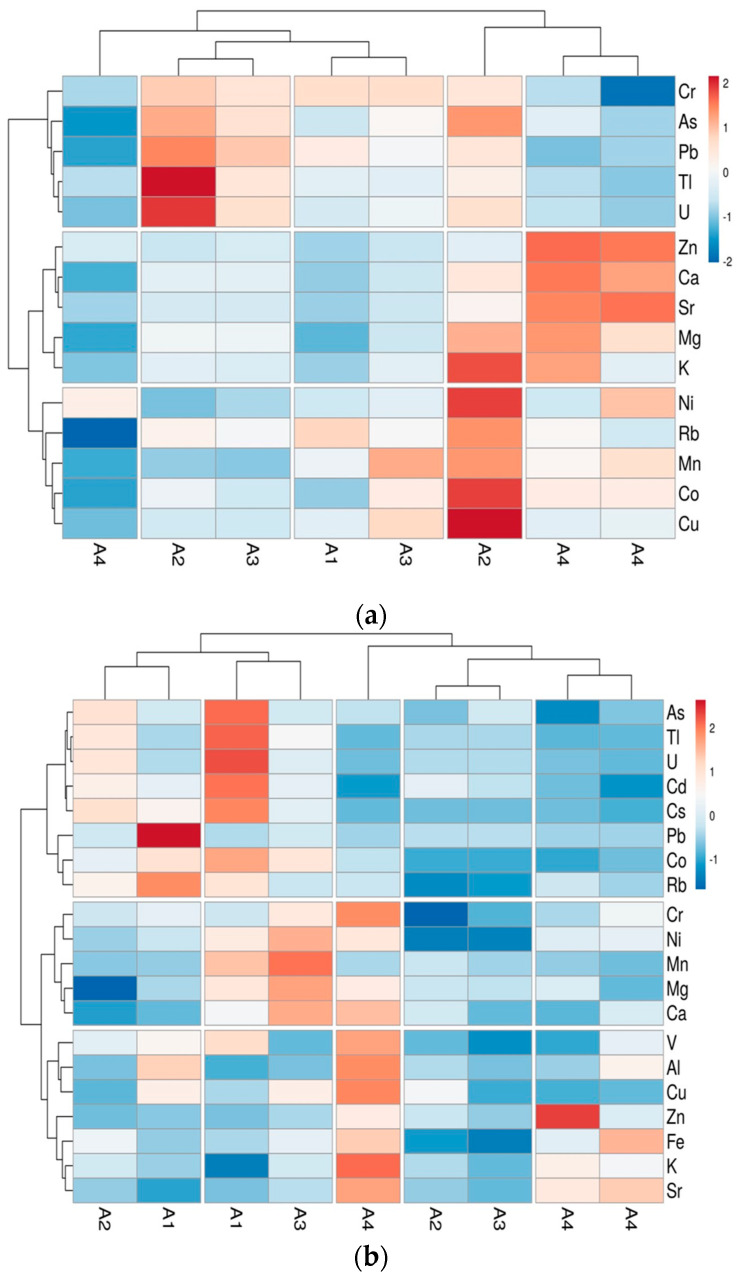
Hierarchical cluster analysis (HCA) of honey (**a**) and bee pollen (**b**) samples.

An aggregative hierarchical cluster analysis (HCA), using Euclidean distances and Ward’s linkage method, was implemented to obtain further data interpretations based on an input matrix consisting of 15 chemical variables (metals) and 17 samples among bee pollen and honey. The results of HCA for honey and bee pollen are shown in the heatmap plot (Figure 12).

Observing the honey HCA (Figure 12a), and in particular the rows, it was possible to highlight three metal groupings, with the first featuring the main heavy metals (Cr, As, Pb, Tl and U), the second being characterized with the macrominerals and some microelements (Ca, Mg, K, Zn, Sr), and the third having Ni, Rb, Mn, Co and Cu. Analysing honey samples, following such groupings, the high content of heavy metals in both the A2 and A3 samples was quite appreciable, while the A4 samples were found to be richer in macrominerals, which are important from a nutritional point of view.

The results for bee pollen HCA were different (Figure 12b). By the row grouping, the first cluster was related to heavy metal except for Cr and Ni, which instead were grouped in the second cluster, followed by the rest of the metals being grouped in the third one. Both the A1 and A2 samples were grouped in regard to heavy metal content, with A1 in particular showing the highest levels of Pb, As and Cd; contrarily, A4 samples proved to poor in terms of the latest elements, except for the presence of Ni and Cr, confirming the wider mobility of bees, and especially in case of flowers scarcity related to meteorological or other adverse conditions giving access to areas wider than 50 km^2^ and therefore coming into contact with more polluted areas [36,97].

The results presented confirmed those previously observed, mainly by PCA analysis. Further, it was quite difficult to directly correlate the presence of metals in bee pollen and honey due to the differences between the two products. Indeed, as frequently mentioned, numerous factors affect the content of metals in beehive products. In particular, it was evident that the effects of bee biotransformation of honey presented a more homogeneous grouping among the samples belonging to the same area than that of bee pollen.

## 4. Conclusions

In the frame of food safety, the multi-elemental profile of honeys and bee pollen provided information regarding both nutritional values and environmental conditions of the harvesting areas of the Abruzzo region. Results reveal differences in the mineral and metal content associated with the influence of biotic and abiotic factors characteristics of each specific area. Negligible values were found for potentially toxic metals such as cadmium, arsenic and lead, which were recovered at concentrations lower than the maximum limit set by European regulations.

Honey can be considered safe for consumption by adults, adolescents and children due to the low carcinogenic and non-carcinogenic risk values. However, there is particular concern for toddlers due to its high LCTRtot value, mainly associated with the accumulation of chromium (Cr) in the product. Despite the high nutritional value, bee pollen exhibited elevated LCTRtot levels in both the adult and children categories, primarily due to the accumulation of lead (Pb) and nickel (Ni), a particular note for attention in regard to for public health. The results also highlighted the relationship between the flight area, well described by the Corine Landcover maps, and the nutritional and safety properties of honey and bee pollen, indicating that the A4 apiary had better results because it was less anthropized.

## Figures and Tables

**Table 1:** Values of parameters used for the assessment of EDI.

Category	Years	Body Weight (BW) (kg)	Average Lifetime (AT) (Days)	Total Exposure (TE) (Years)	Intake Rate (IR) Honey ^A^ (kg/Day)	Intake Rate (IR) Bee Pollen ^B^ (kg/Day)
toddler	0–3	11.3	730	2	0.0127	-
children	3–10	26.1	2555	7	0.0126	0.02
adolescent	10–18	52.6	2920	8	0.0133	-
adult	18–65	69.7	17155	47	0.0127	0.04

Bibliographic references for values used in this study: ^A^ [2,54,55,56]; ^B^ [11,27,52,56,57,58,59].

**Table 2:** Oral reference dose (mg/kg_bw_/day) and cancer slope factor (mg/kgbw/day)^−1^ for each element.

Elements	RfD (mg/kg_bw_/Day)	Reference	CSF (mg/kg_bw_/Day)	Reference
		[53]		/
Cd	0.0001	[51]	0.38	[62,64,65]
V	0.005 **	[51]	/	/
Cr	0.003 ***	[51]	0.50	[66]
Ni	0.02 ▴	[51]	1.70 ◦	[66]
Cu	0.04	[51]	/	/
As	0.0003 ▴▴	[51]	1.50	[51]
Ba	0.20	[51]	/	/
Sb	0.0004 ▴▴▴	[51]	/	/
Pb	0.0035	[60,61,62,63]	0.0085 ◦◦	[66]
Mn	0.10	[51]	/	/
Al	1	[67]	/	/
Fe	0.7	[68]	/	/
Co	0.0003	[67]	/	/
Rb	0.005	[69]	/	/
Zn	0.3	[67]	/	/
U	0.003	[70]	/	/

** Vanadium and Compounds, *** Chromium VI, ▴ Nickel Soluble Salts, ▴▴ Inorganic Arsenic, ▴▴▴ Antimony (metallic) and Antimony Tetroxide, ◦ Nickel Subsulfide, ◦◦ Lead and Compounds. Adapted from [2].

**Table 3:** Elemental composition of honeys (μg g^−^^1^ ± standard deviation (*n* = 3)).

Element	A1 2020	A2 2019	A2 2020	A3 2019	A3 2020	A4 2019	A4 2019	A4 2020
Mg	10.875 ± 0.339	20.016 ± 1.163	29.496 ± 0.509	15.703 ± 0.940	19.547 ± 0.634	31.22 ± 0.849	9.584 ± 0.387	25.438 ± 0.175
Al	0.351 ± 0.042	n.d	2.314 ± 0.251	1.485 ± 0.066	n.d.	0.607 ± 0.021	0.117 ± 0.017	0.188 ± 0.008
K	445.962 ± 2.448	542.353 ± 20.797	885.447 ± 27.102	547.545 ± 6.164	532.569 ± 23.354	797.00 ± 3.030	423.783 ± 0.553	549.793 ± 5.435
Ca	22.155 ± 1.091	35.103 ± 1.269	48.314 ± 0.591	28.199 ± 1.977	34.665 ± 0.525	69.32 ± 0.465	15.217 ± 0.317	63.533 ± 0.964
V	0.001 ± 0.000	0.001 ± 0.000	0.005 ± 0.000	0.003 ± 0.000	0.001 ± 0.001	0.001 ± 0.000	n.d	0.001 ± 0.000
Cr	0.238 ± 0.008	0.243 ± 0.008	0.234 ± 0.004	0.238 ± 0.006	0.235 ± 0.008	0.205 ± 0.003	0.203 ± 0.001	0.176 ± 0.004
Mn	0.230 ± 0.008	0.131 ± 0.007	0.412 ± 0.011	0.388 ± 0.013	0.126 ± 0.009	0.261 ± 0.000	0.085 ± 0.001	0.320 ± 0.002
Fe	n.d	n.d	n.d	n.d	n.d	n.d	n.d	n.d
Co	0.001 ± 0.000	0.002 ± 0.000	0.004 ± 0.000	0.003 ± 0.000	0.002 ± 0.000	0.003 ± 0.000	0.001 ± 0.000	0.003 ± 0.000
Ni	0.019 ± 0.000	0.015 ± 0.002	0.038 ± 0.004	0.021 ± 0.003	0.017 ± 0.001	0.019 ± 0.001	0.026 ± 0.000	0.031 ± 0.000
Cu	0.206 ± 0.003	0.173 ± 0.010	0.564 ± 0.009	0.361 ± 0.019	0.167 ± 0.007	0.209 ± 0.005	0.091 ± 0.000	0.222 ± 0.005
Zn	0.157 ± 0.002	0.364 ± 0.046	0.778 ± 0.023	0.373 ± 0.056	0.639 ± 0.014	2.956 ± 0.095	0.602 ± 0.009	2.826 ± 0.067
As	0.003 ± 0.001	0.006 ± 0.001	0.006 ± 0.001	0.004 ± 0.000	0.005 ± 0.001	0.004 ± 0.001	0.002 ± 0.001	0.003 ± 0.000
Rb	0.499 ± 0.003	0.429 ± 0.017	0.567 ± 0.020	0.412 ± 0.027	0.408 ± 0.015	0.415 ± 0.027	0.175 ± 0.003	0.349 ± 0.004
Sr	0.081 ± 0.001	0.131 ± 0.008	0.205 ± 0.001	0.116 ± 0.009	0.131 ± 0.001	0.356 ± 0.005	0.086 ± 0.002	0.367 ± 0.006
Cd	n.d	0.0003 ± 0.0002	n.d	n.d	n.d	n.d	n.d	n.d
Cs	0.000 ± 0.000	0.001 ± 0.000	0.001 ± 0.000	0.000 ± 0.000	0.001 ± 0.000	0.000 ± 0.000	n.d	0.000 ± 0.000
Tl	0.001 ± 0.000	0.002 ± 0.000	0.001 ± 0.000	0.001 ± 0.000	0.001 ± 0.000	0.001 ± 0.000	0.001 ± 0.000	0.000 ± 0.000
Pb	0.039 ± 0.001	0.064 ± 0.003	0.002 ± 0.000	0.032 ± 0.002	0.052 ± 0.002	0.009 ± 0.001	0.001 ± 0.001	0.013 ± 0.000
U	0.000 ± 0.000	0.001 ± 0.000	0.000 ± 0.000	0.000 ± 0.000	0.001 ± 0.000	0.000 ± 0.000	0.000 ± 0.000	0.000 ± 0.000

n.d: not detected.

**Table 4:** Elemental composition of polyfloral bee pollen (μg g^−^^1^ ± standard deviation (n = 3)).

Element	A1	A1	A2	A2	A3	A3	A4	A4	A4
Mg	976 ± 104	798 ± 60	621 ± 1	819 ± 6	1089 ± 36	813 ± 35	857 ± 58	962 ± 47	751 ± 55
Al	4 ± 1	25 ± 2	6 ± 1	9 ± 1	6 ± 1	6 ± 1	8 ± 1	31 ± 1	18 ± 1
K	5185 ± 412	5711 ± 399	5930 ± 23	5787 ± 135	5929 ± 82	5543 ± 143	6370 ± 453	7186 ± 261	6229 ± 273
Ca	1328 ± 74	1066 ± 87	1001 ± 8	1212 ± 71	1591 ± 38	1067 ± 24	1059 ± 51	1549 ± 71	1231 ± 48
V	0.08 ± 0.02	0.07 ± 0.01	0.06 ± 0.05	0.04 ± 0.01	0.04 ± 0.03	0.03 ± 0.01	0.04 ± 0.01	0.09 ± 0.01	0.06 ± 0.01
Cr	0.18 ± 0.04	0.20 ± 0.01	0.18 ± 0.06	0.11 ± 0.02	0.23 ± 0.04	0.15 ± 0.02	0.17 ± 0.01	0.28 ± 0.01	0.21 ± 0.01
Mn	71 ± 3	24 ± 1	23 ± 1	32 ± 2	87 ± 4	27 ± 0.4	24 ± 1	28 ± 1	20 ± 1
Fe	60 ± 2	56 ± 4	82 ± 8	39 ± 2	78 ± 4	29 ± 1	76 ± 2	113 ± 3	120 ± 11
Co	0.10 ± 0.07	0.08 ± 0.01	0.06 ± 0.05	0.03 ± 0.01	0.08 ± 0.03	0.03 ± 0.01	0.03 ± 0.01	0.05 ± 0.004	0.04 ± 0.003
Ni	1.89 ± 0.01	1.07 ± 0.04	0.88 ± 0.09	0.14 ± 0.02	2.50 ± 0.20	0.18 ± 0.02	1.31 ± 0.01	1.96 ± 0.02	1.40 ± 0.10
Cu	12.3 ± 0.5	14.1 ± 0.7	11.7 ± 0.6	13.6 ± 0.8	14.1 ± 0.8	11.5 ± 0.2	11.6 ± 0.2	16.0 ± 0.2	11.8 ± 0.2
Zn	47.0 ± 0.4	51 ± 1	44 ± 3	65 ± 4	57 ± 3	53 ± 1	179 ± 8	109 ± 4	76 ± 2
As	0.09 ± 0.10	0.05 ± 0.02	0.07 ± 0.07	0.04 ± 0.02	0.05 ± 0.05	0.05 ± 0.02	0.03 ± 0.01	0.05 ± 0.01	0.04 ± 0.001
Rb	7.71 ± 0.07	9.70 ± 0.50	6.90 ± 0.70	2.60 ± 0.20	4.90 ± 0.40	3.00 ± 0.10	5.07 ± 0.10	4.90 ± 0.03	4.42 ± 0.04
Sr	1.90 ± 0.20	1.22 ± 0.001	2.10 ± 0.20	2.10 ± 0.10	2.60 ± 0.20	1.70 ± 0.09	4.87 ± 0.04	6.55 ± 0.01	5.78 ± 0.06
Cd	0.08 ± 0.10	0.04 ± 0.01	0.05 ± 0.06	0.04 ± 0.02	0.04 ± 0.04	0.03 ± 0.02	0.02 ± 0.01	0.01 ± 0.01	0.01 ± 0.003
Cs	0.08 ± 0.09	0.05 ± 0.01	0.06 ± 0.06	0.02 ± 0.01	0.04 ± 0.04	0.02 ± 0.02	0.02 ± 0.009	0.02 ± 0.01	0.02 ± 0.002
Tl	0.08 ± 0.09	0.02 ± 0.01	0.05 ± 0.06	0.02 ± 0.02	0.04 ± 0.04	0.02 ± 0.02	0.01 ± 0.01	0.01 ± 0.01	0.01 ± 0.003
Pb	0.40 ± 0.10	13.80 ± 0.70	1.40 ± 0.20	0.66 ± 0.06	1.70 ± 0.20	0.64 ± 0.04	0.05 ± 0.01	0.06 ± 0.01	0.05 ± 0.02
U	0.08 ± 0.09	0.02 ± 0.01	0.05 ± 0.06	0.02 ± 0.02	0.03 ± 0.04	0.02 ± 0.02	0.01 ± 0.010	0.01 ± 0.007	0.01 ± 0.003

## Data Availability

The original contributions presented in the study are included in the article/Supplementary Materials; further inquiries can be directed to the corresponding author.

## Supplementary Materials

The following supporting information can be downloaded at: https://www.mdpi.com/article/10.3390/foods13121930/s1, Figure S1a: Contribution (%) of each metal to HI due to the consumption of honey, Figure S1b: Contribution (%) of each metal to HI due to the consumption of bee pollen; Table S1: Description of the codes was reported in Supplementary Materials; Table S2: ICP-MS instrumentation and operating conditions; Table S3: Elemental composition of polyfloral honey found in the present research (Abruzzo) and those found in the literature (μg g^−1^); Table S4: Elemental composition of polyfloral bee pollen found in the present research (Abruzzo) and those found in the literature (μg g^−1^).
