# Supplementary material for: Assessing Mineral Content and Heavy Metal Exposure in Abruzzo Honey and Bee Pollen from Different Anthropic Areas
Source: Foods. 2024 Jun 19;13(12):1930. doi: 10.3390/foods13121930 (PMC11202465; doi:10.3390/foods13121930)
Supplement: Supplementary file 1 [file foods-13-01930-s001.zip › foods-3025747-supplementary.pdf]

Table S1. Description of the codes was reported in Supplementary materials

CODE	DESCRIPTION
111	Continuous urban fabric: when urban structures and transport networks are dominating the surface area. >80% of the land surface is covered by impermeable features like buildings, roads, and artificially surfaced areas. Non-linear areas of vegetation and bare soil are exceptional
112	Discontinuous urban fabric: when urban structures and transport networks associated with vegetated areas and bare surfaces are present and occupy significant sur-faces in a discontinuous spatial pattern
211	Non-irrigated arable land: cultivated land parcels under rainfed agricultural use for annually harvested non-permanent crops, normally under a crop rotation system, including fallow lands within such crop rotation. Fields with sporadic sprinkler-irrigation with non-permanent devices to support dominant rainfed cultivation are included
221	Vineyards: areas planted with vines, vineyard parcels covering >50%, and determining land use. The impermeable features like buildings, roads and artificially surfaced areas range from 30 to 80 % land coverage
242	Complex cultivation patterns: mosaic of small cultivated land parcels with different cultivation types - annual crops, pasture and/or permanent crops, eventually with scattered houses or gardens.
243	Land principally occupied by agriculture, with significant areas of natural vegetation
244	Agro-forestry areas: annual crops or grazing land under the wooded cover of forestry species.
311	Broad-leaved forest: vegetation formation composed principally of trees, including shrub and bush understorey, where broad-leaved species predominate
523	Sea and ocean: zone seaward of the lowest tide limit

Table S2. ICP-MS instrumentation and operating conditions

**Instrument parameters**	**(Agilent 7500A, Agilent Technologies, Tokyo, Japan)**
Nebulizer	Babington
Torch	Quartz glass torch
Spray chamber	Scott double-pass type at 2 °C
Sample cone	Nickel, 1.00 mm aperture
Skimmer cone	Nickel, 0.40 mm aperture
Plasma mode	Normal plasma
RF power (W)	1240
Sampling depth	8.8 mm
Nebulizer gas	1.07 L/min
Plasma gas	15 L/min
Sampling period (s)	0.3

Integration time (s)	0.1
Repetitions	3
Sample uptake rate	0.4 mL/min

Table S3. Elemental composition of polyfloral honey found in the present research (Abruzzo) and those found in the literature (μg g^{-1} ± standard deviation (n=3)).

	**Abruzzo**	**Lazio**	**Sicilia**	**Lazio[a]**	**Umbria**	**South Italy**	**South Italy**	**South Italy**	**Marche**	**Campani**	**Basilicata**	**Piemonte**	**Italia**	**Centre Italy**	**MAX**	**MIN**
**Mg**	20.24		8.17	23.0							19.3				69	8
**K**	590.56		168.80	347.0				1610.0							1610	56
**Ca**	39.56		26.10	77.0			68	176.0			44.1				206	6.64
**Cr**	0.222	0.08	0.11	<0.04	0.27	0.71	0.01	0.26	0.009	0.07	0.02	0.078	0.0190	0.072	2.19	0.00158
**Mn**	0.244			0.20	0.87		0.66	1.34		0.88	1.60	1.920	6.10		9.02114	0.049
**Co**	0.002			<0.004			0.00396				0.004	0.008	0.0083		0.0151	0.00074
**Ni**	0.023	0.1		<0.6				0.13	0.039	0.17	0.03	0.168	0.0370		0.321	0.01495
**Cu**	0.249		1.98	<0.4	0.39		0.22	0.26		1.05	0.24	0.632	0.9100	0.300	1.98	0.02642
**Zn**	1.087	3.00	2.03	<1	0.30		1.07	3.45			1.08	1.20	1.50		11.23	0.1
**As**	0.004	0.03		<0.03			0.001			0.015	0.001	0.013	0.0061		0.03	0.0001
**Rb**	0.407			4.49								8.500	16.0		23	0.006
**Sr**	0.184			0.3			0.232				0.115				1.557	0.0248
**Tl**	0.001			0.0007			0.032								0.535	0.00002
**Pb**	0.032	0.03	0.171	0.011	0.18	0.289	0.033	0.04	0.00	0.030	0.0111	0.036	0.0140	0.042	1.39	0.00085

**U**	0.001	0.0003		0.0003			0.0001								0.0009	0.00001
**Cd**		0.003		0.0007	0.02	0.013	0.0006		0.00	0.006	0.0033			0.005	0.27	0.00017
**Hg**		0.0021		0.0008	0.07		0.0002			0.881					1.713	0.0004
**Ref**	*	[48]	[74]	[75]	[83]	[79]	[76]	[77]	[42]	[2]	[18]	[16]	[40]	[84]		

* present research

Table S4. Elemental composition of polyfloral bee pollen found in the present research (Abruzzo) and those found in the literature (μg g^{-1} ± standard deviation (n=3)).

	**Abruzzo**	**Lazio**	**Lazio**	**Swiss**	**Turky**	**Review**
**Mg**	854		483	200-3000	648–2150	200-3600
**Al**	12.6		24.1			
**K**	5985.5		4880	4000-20000		
**Ca**	1233.7		973	200-3000	726–2202	180-6500
**V**	0.0563		0.08			
**Cr**	0.18	0.22	0.12		0,85-3,4	0.002–42
**Mn**	37.3		26	20-110	18-117	5.1–430
**Fe**	72.6		42.7	11-170	35–811	6.6–1180
**Co**	0.05		0.044			

**Ni**	1.25	0.3	<0,7		2.32-21.7	0,002-6,85
**Cu**	12.9		6.2	2-16	8–26	0.11–27.7
**Zn**	75.6	27	23	30-250	18-48	5.1–162
**As**	0.05	0.05	<0,2		1.3–2.25	0.007-14,71
**Rb**	5.46		30.1			
**Sr**	3.20		3.9			
**Cd**	0.03	0.033	0.021		0.039–1.39	0,0016-15,40
**Cs**	0.03		0.065			
**Tl**	0.02		0.0091			
**Pb**	2.08	0.12	0.08		0.005–0.622	0,001-18,2
**U**	0.02	0.0057	0.026			
**Hg**		0.0067	0.0008			
**Ref**	*	[48]	[74]	[84]	[27]	[58]

* present research
