# Supplementary material for: Assessing Mineral Content and Heavy Metal Exposure in Abruzzo Honey and Bee Pollen from Different Anthropic Areas
Source: Foods. 2024 Jun 19;13(12):1930. doi: 10.3390/foods13121930 (PMC11202465; doi:10.3390/foods13121930)
Supplement: Supplementary file 1 [file foods-13-01930-s001.zip › Supplementary Figure S1.pptx]

## Slide 1
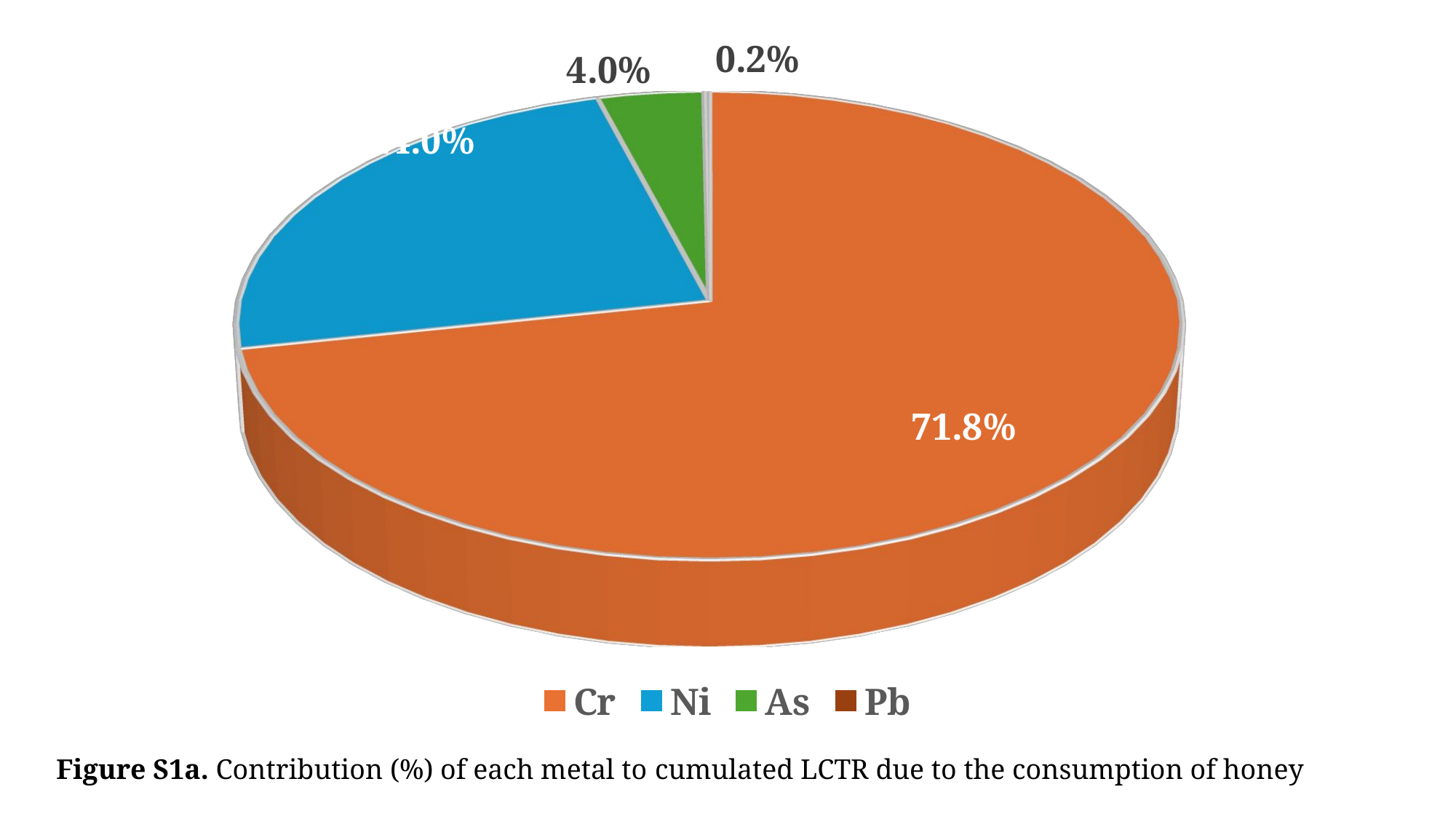

[unsupported chart]
Figure S1a. Contribution (%) of each metal to cumulated LCTR due to the consumption of honey

## Slide 2
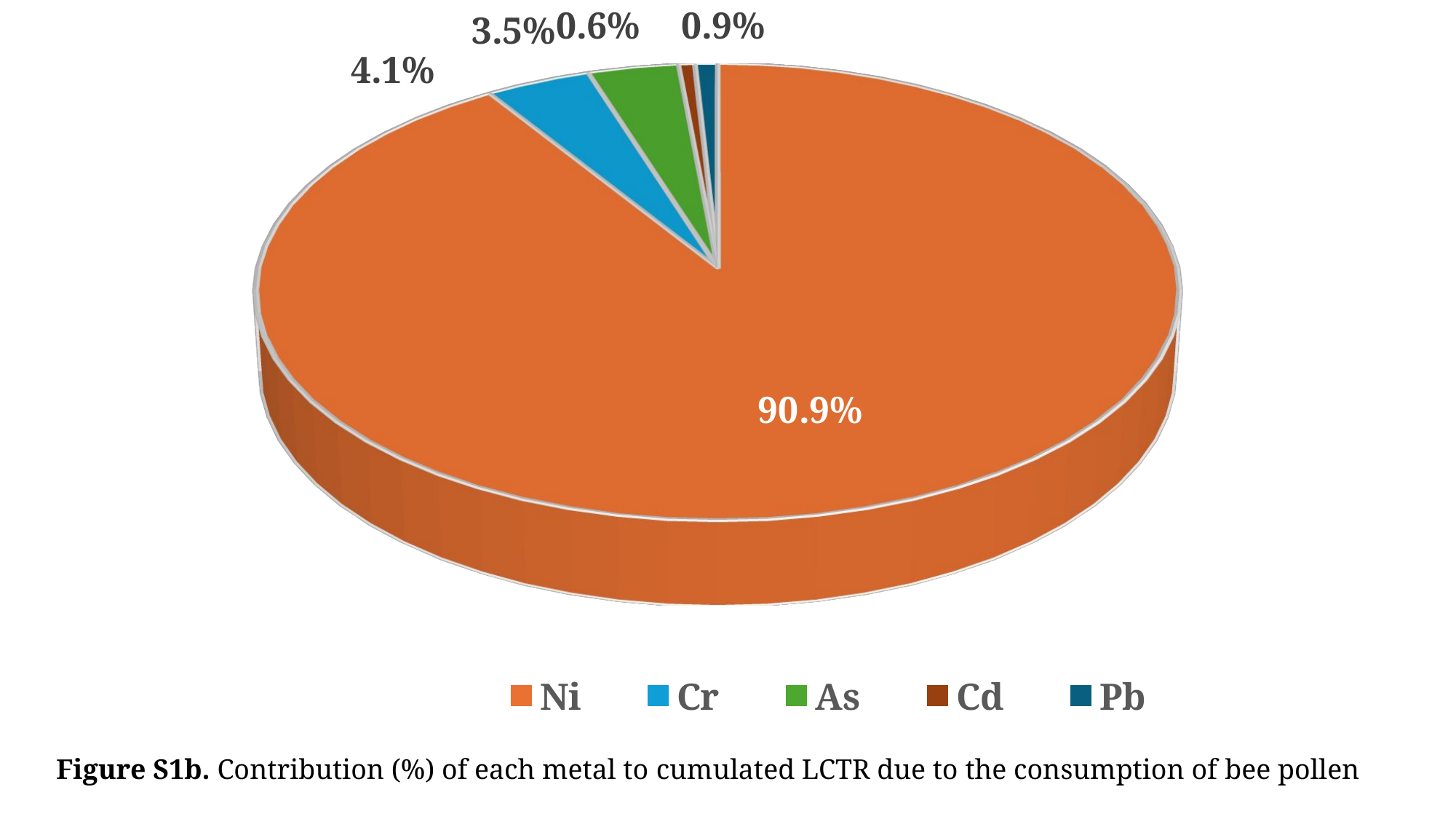

[unsupported chart]
Figure S1b. Contribution (%) of each metal to cumulated LCTR due to the consumption of bee pollen
